# Design and Validation of a Questionnaire to Measure Patient Experience in Relation to Hospital Nursing Care

**DOI:** 10.3390/nursrep14010031

**Published:** 2024-02-09

**Authors:** Nieves López-Ibort, Angel Boned-Galán, Miguel Cañete-Lairla, Carmen Angustias Gómez-Baca, Marina Angusto-Satué, Josep-Oriol Casanovas-Marsal, Ana Gascón-Catalán

**Affiliations:** 1 Hospital Clínico Universitario Lozano Blesa, 50009 Zaragoza, Spain; nlopezi@salud.aragon.es (N.L.-I.); cagomez@salud.aragon.es (C.A.G.-B.); 2 Instituto de Investigación Sanitaria de Aragón, 50009 Zaragoza, Spain; acboned@salud.aragon.es (A.B.-G.); jocasanovas@iisaragon.es (J.-O.C.-M.); 3 Departamento de Fisiatría y Enfermería, Facultad de Ciencias de la Salud, Universidad de Zaragoza, 50009 Zaragoza, Spain; 4 Hospital Universitario Miguel Servet, 50009 Zaragoza, Spain; 5 Departamento de Psicología y Sociología, Facultad de Educación, Universidad de Zaragoza, 50009 Zaragoza, Spain; mcanete@unizar.es; 6 Hospital de Barbastro, 22300 Huesca, Spain

**Keywords:** patient experience, questionnaire, validation, nursing, hospital

## Abstract

The objective has been to develop and validate a questionnaire to know patient experience in relation to nursing care during their hospital stay in the Spanish healthcare setting. To know patient experience will improve the quality of care of the healthcare system; therefore, we must count on validated tools so it can be evaluated in an accurate way. Method: a questionnaire containing 29 items alongside socio-demographic questions was developed. It was distributed to 158 patients admitted to a tertiary hospital. The psychometric properties were assessed through principal components analysis and confirmatory factor analysis to evaluate construct validity, employing Cronbach’s alpha to test reliability. Results: The final tool contains 17 items grouped into 5 dimensions: interrelations, nursing care, information during hospital stay, information about patient’s rights, and discharge information. Two additional questions related to pain were added. The questionnaire showed adequate validity and reliability. Conclusions: we describe a new tool validated and adapted to the Spanish healthcare setting with adequate validity and reliability to assess patient experience with nursing professionals during hospital stay. This tool will serve to identify areas for improvement in hospital nursing care and as an instrument in the management and supervision of nursing teams.

## 1. Introduction

In the healthcare system, hospitals play an essential role in healthcare when the effect of the activities they offer, challenges they have to face, and the existing limited resources are considered. The evolution of healthcare professional–patient relationships and the rise of chronic diseases require greater integration of patients into their healthcare pathways. In fact, patients must continue the treatment on their own, be capable of adapting it, and even be aware of warning signs related to their illnesses. In healthcare, the integration of patient experiences promotes new perspectives that transcend traditional healthcare boundaries. However, there is still a lack of empirical studies describing these patient experiences to design new forms of collaboration and organization [1].

Patient experience is considered a key factor in the quality of healthcare as patient experience high scores are related to a higher clinical quality [2]. For the National Health Service, the quality of care is based on three domains: patient safety, clinical effectiveness, and patient experience, which are interrelated, with a positive association existing among them [3]. Patient experience is defined as the sum of all interactions, shaped by an organization’s culture and the influence of patient perception across the continuum of care, from their first contact until their discharge [4]. There is a disconnection between what patients want, what care teams believe patients want and what hospital setting offers [5]. In this regard, Bezos indicates that enhancing the patient experience involves listening to the deep needs of patients and, together with them, transforming the healthcare context in order to achieve health and well-being results that could be scientifically measured [6].

Improving patient experience does not lie in demanding unnecessary care services; it does require, however, a clear communication with patients about the reason for which the care given is the most appropriate and in their best interest [7]. From this patient–health professional communication, hospitals may draw information to focus on specific areas of improvement, strategic decision making, patient expectations management, and assessment. From the patient experience, some recommendations may be drawn in order to strengthen the quality of healthcare, promote communication, improve infrastructures, and optimize how to carry out patient monitoring outside hospital setting.

Nurses are probably the ones who have more influence on patient experience due to the amount of time spent on care giving and to the way they interact and communicate with patients. Existing research generally points out the relation between nurse staffing and patient care experience [8,9]. From a qualitative point of view, some potential barriers to improve patient experience have been revealed, such as workload, staffing limitations, uncontrollable environmental conditions, and unrealistic patient expectations.

Patient experience does not equate to the satisfaction derived solely from health assistance provided to the patient. The difference between satisfaction and experience is important, as satisfaction-related questions for the patient may be more prone to subjectivity and that makes it more difficult to know how to implement improvements. In contrast to satisfaction surveys, experience surveys collect data pertaining to whether behaviors deemed important by the patient are occurring and their frequency. Experience surveys are more objective and capable of reporting about adequate actions for improvement. Patient-centered-care improvement actions are a priority, and there are many on-going initiatives with variable success [10].

There is a range of instruments that have been used to evaluate patient’s perceptions about hospital care. Survey tools have been developed in order to measure patients’ vision in different units, including the general hospital care, radiology, pediatric units, elderly care, psychiatry unit, chronic patients, and many others. Most user-perception surveys about healthcare services include general questions about information and participation. The problem with general surveys is that it is difficult to disaggregate results to determine, for example, the different behavior of different units, different professionals, different clinical situations, etc.

**Figure 1:**
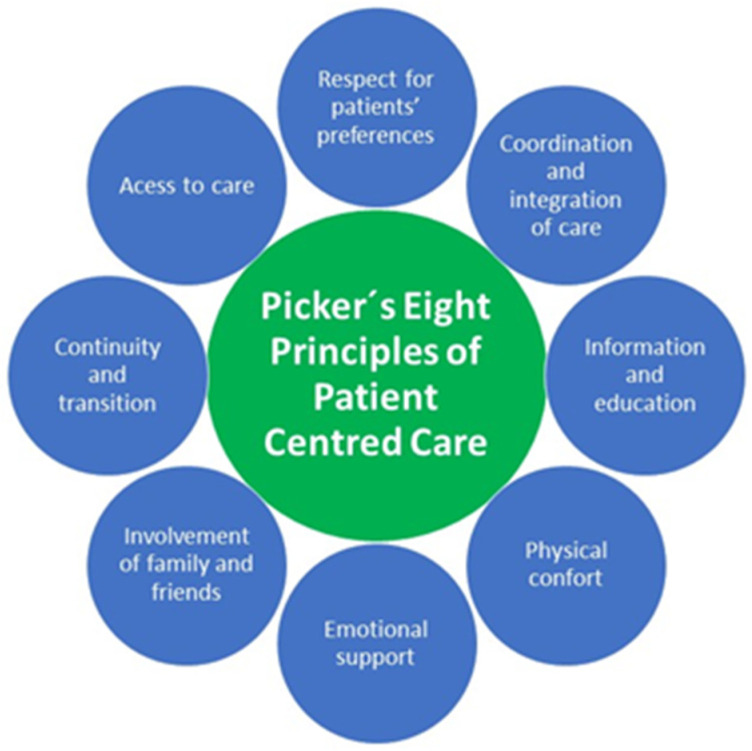
Patient experience framework.

In the Anglo-Saxon literature there are tools, such as in the Picker Patient Experience Questionnaire, in which only some questions address patient experience with nursing staff [11]. This questionnaire is based on these 8 standards: respect for patients’ preferences, coordination and integration of care, information and education, physical comfort, emotional support, involvement of family and friends, continuity and transition, and access to care. These values have also been proposed by the Harvard Medical School and The Commonwealth Fund, and they represent the framework of this study (Figure 1).

So far, there is no questionnaire specifically designed to evaluate how patients perceive health services regarding nursing care. Therefore, it seems appropriate to have tools aimed at, in a more specific way, exploring the different components of the information process, consent, and decision-making right, as they are internalized by patients in relation to nursing care [12]. Therefore, our goal is to validate a patient experience questionnaire regarding nursing care during hospital stay.

## 2. Materials and Methods

### 2.1. Study Design

Development of a questionnaire based on the Picker questionnaire and its validation in order to evaluate nursing care patient experience during hospital stay.

### 2.2. Participants

Patients who have been hospitalized at Miguel Servet Hospital in Medicine or Surgery Units have been discharged from 15 January 2020 to 13 March 2020.

Inclusion criteria: adults aged 18 years and older admitted to inpatient units with a duration exceeding 24 h.

Exclusion criteria: patients from emergency room and psychiatry units, cognitive impairment that hinder patients from understanding the questionnaire, not being able to understand the language, and rejection to sign informed consent.

For the sample size calculation, we followed the recommendations from the current literature, which suggests the need for a minimum of 5 subjects per questionnaire item [13,14]. This enables the execution of psychometric calculations for validity and reliability with statistical significance and representativeness.

Participants were selected aleatorily and proportionally to the number of admissions in each unit in a year. The unit nurse manager reported daily to the research group the patients that were discharged. Questionnaires were distributed by nursing professionals before the patients left the room. The response rate was 98.13%. The information gathering took place from 15 January 2020 to 13 March 2020.

### 2.3. Procedure

For the development of the questionnaire, the participation of expert professionals from various fields (nursing, medicine, and psychology) was sought. A schedule of regular meetings was established, during which, after individually reviewing the diverse literature, items from the Picker Patient Experience Questionnaire [11], other items identified in the literature, and those based on aspects primarily related to nursing care were selected.

**Figure 2:**
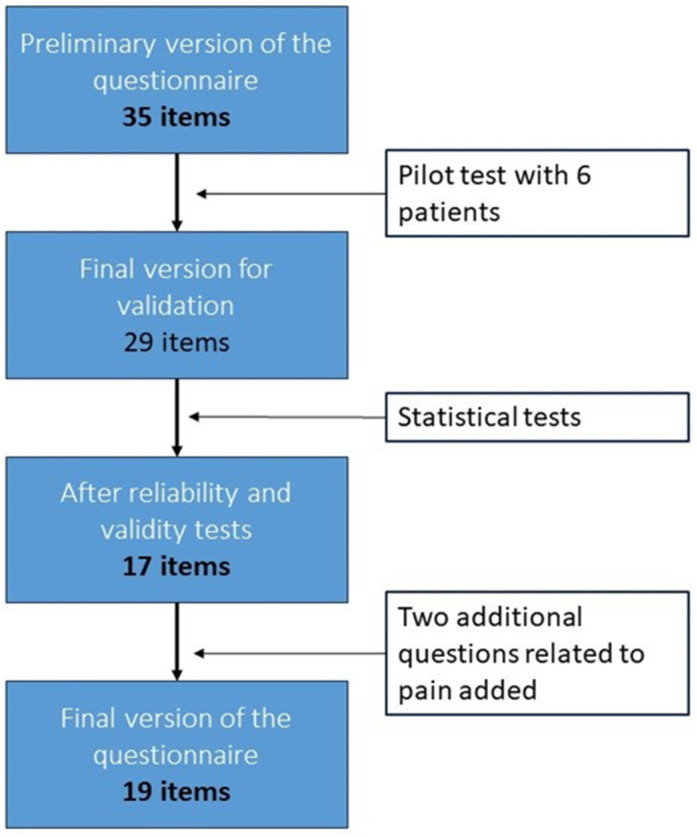
Items selection process.

To design the questionnaire, the experts group selected and adapted some items from the Picker questionnaire and added others from the consulted literature, resulting in a 35-item questionnaire (Figure 2). Once a preliminary version of the questionnaire was obtained, a pilot test was conducted. It was administered jointly by two nurses to 6 patients of varying ages. Various aspects were recorded, such as the time taken to complete the questionnaire, the patient’s understanding of each item, clarity and relevance of items, measure affected by social desirability bias, high endorsement of a single option, if respondents fail to complete an item, researcher’s interpretation of patient comments, and comments related to the process. The expert group that reviewed these data allowed the reformulation of the wording for some items and the elimination of duplicates and those with poorer comprehension by the surveyed individuals, including those items deemed theoretically important. Thus, 6 items were eliminated resulting in the final version comprised 29 items for validation (Figure 2, File S1).

Once the final version of the questionnaire was drawn up, patients were contacted in person in the hospital on the day of discharge in their room or in the discharge area or in their first visit to the out-patient department after their hospital discharge. The nurse explained the goals of the study and asked for their engagement and for the signing of the informed consent. Questionnaire data regarding hospital stay were obtained from discharge report and users data base. For the reproducibility test–retest study, the questionnaire was distributed by phone to a subsample after one month from hospital discharge.

### 2.4. Instrument

The questionnaire consisted of questions selected from the Picker’s Patient Experience and was geared to evaluate patient experience with nursing professionals during hospital stay and questions considered relevant by the experts for the assumed goal.

### 2.5. Data Analysis

#### 2.5.1. Descriptive Analysis of the Sample

A descriptive analysis of the studied variables was conducted. Qualitative variables were presented as percentages and frequencies and quantitative variables were expressed using measures of central tendency (mean) and dispersion (standard deviation, minimum, maximum).

#### 2.5.2. Validation Analysis

As statistic exclusion criteria, questionnaires that had not been completed correctly were excluded.

To check the goodness of the scale a reliability and validity study from different perspectives will be carried out. Regarding reliability we will measure, in the first place, concordance between answers given at patient’s discharge and answers collected by phone one month after discharge, thus the test–retest agreement coefficient is calculated through Cohen’s Kappa coefficient. The scale internal consistency will be given by the Cronbach alpha coefficient. Calculation of such a coefficient will be conducted in conjunction with the items’ analysis through their discrimination indexes, allowing us to discard those items that do not overtake the minimum established values for those indexes, that is, they do not properly discriminate.

Regarding validity, experts on patient experience checked, during scale elaboration, that the scale consisted of representative items of the measurement construct and proportionally to the importance of the different components, that is, to the content validity. Once the scale is analyzed for its reliability calculation and those items with low ability to discriminate have been removed, we will check the construct validity using a Confirmatory Factor Analysis (CFA), checking that the dimensions of the scale studied previously with the Exploratory Factor Analysis (EFA) have a proper adjustment through the structural equation method.

For all these statistical indexes calculations we used the statistical package IBM SPSS 24 except for the CFA where the statistical package Lisrel 8.80 was used.

### 2.6. Ethical Considerations

All individuals who chose to participate received oral information in addition to the informed consent document to be signed. Nurses were who, before handing the questionnaire, provided the informed consent, answered any queries and facilitated time to read it and sign it. Informed consent in research involves ensuring participants provide consent freely without coercion, transparent disclosure of information, comprehension, ongoing communication, and the right to withdraw. Additionally, privacy and confidentiality must be maintained. To ensure the anonymity of the survey respondents, each questionnaire was assigned a unique registration code, linking the signed informed consent with the collected data. Only the person responsible for administering the questionnaire, and if applicable, for making the telephone call for the retest, knew the name of the surveyed individual. Patient-identifying data were not transferred to the spreadsheet used for subsequent statistical analysis, only the assigned identifier was included. Approval for the study was obtained from the Clinical Research Committee of Aragon (PI19/35).

## 3. Results

### 3.1. Sample Description

The sample consisted of 158 patients ranging from 19 to 93 years old with an average age of 66.61 (SD 17.95 years old) and 50% women. Most of the patients had primary education (44.9%). A total of 62% were retired (Table 1).

### 3.2. Test–Retest Measures Concordance

For test–retest concordance, the Cohen’s Kappa coefficient is calculated for each and every of the 29 questions of the patient experience scale (Table 2).

The responses given to question 19 in the retest were identical for all patients, so Kappa could not be calculated for this item. In 16 of the 28 questions (57%), question 19 excluded, significant agreement values are reached. The test–retest correlation showed a good temporal consistency of the patients answers.

### 3.3. Reliability

Regarding the test–retest reliability, Kendall’s tau-b correlation coefficient for ordinal variables between the two implementations of the test, shows a value of 0.516 with a significance of *p* < 0.001, what means a sufficiently high value.

The discrimination indices for all items, excluding those with an item–test correlation value below 0.20 according to the Likert criterion, result in a scale composed of 22 items with an internal consistency coefficient α = 0.792. It can be asserted that the 22-item scale has good reliability. The eliminated items were 1, 3, 7, 13, 16, 19, and 26.

### 3.4. Validity

The dimensions underlying the patient experience scale were examined through Exploratory Factor Analysis (EFA). The Kaiser–Meyer–Olkin measure of sampling adequacy is 0.849, with a χ^2^ value of 559.741 and a significance level of *p* < 0.001 for the Bartlett’s sphericity test, both indicating the suitability of using Factor Analysis. Five dimensions or factors are postulated, explaining 53% of the total variance with 22 items. The Principal Axis Factoring extraction method was employed due to the lack of fit to normality, and the Promax oblique rotation method with Kaiser normalization was used due to the presumed relationships between factors. The simplified factorial matrix is shown in Table 3.

The study of the factorial matrix, along with the previous item analysis and corresponding theoretical support, allowed us to propose the following dimensions with their associated items:

1. Interrelationships: 11, 22, and 25;
2. Nursing care: 2, 4, 5, 14, and 27;
3. Information during hospital stay: 6, 10, and 12;
4. Patient rights information: 17 and 24;
5. Discharge Information: 15, 21, 23, and 28.

This totals 17 items. The remaining items were eliminated through various item selection techniques. The structure was tested using Confirmatory Factor Analysis (CFA) through the Structural Equation Modeling (SEM) method. Since the item responses are ordinal with five or fewer categories, the Diagonal Weighted Least Squares (DWLS) method was used. Table 4 displays the goodness-of-fit statistics for the model.

**Figure 3:**
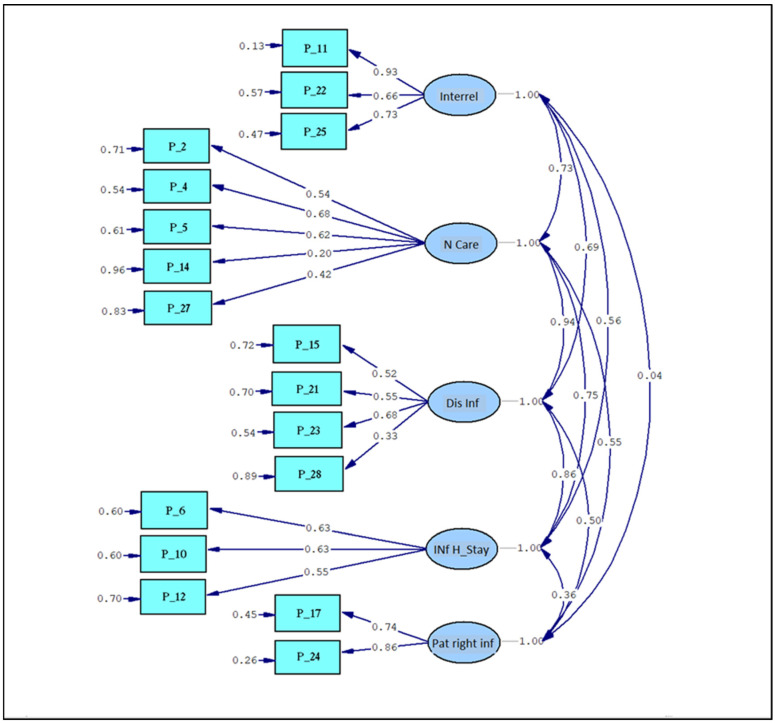
Graphical representation and regression/correlation values. Note: interrel = interrelationships; N Care = nursing care; Dis Inf = discharge information; Inf H Stay = information during hospital stay; Pat right inf = patient’s rights information.

Out of the ten indicators, seven reported that the fit to the proposed model is good, while the other three indicate an acceptable fit. None of these indicators report a poor fit. The validated structure is shown in Figure 3.

The arrows on the right side of the figure indicate the intercorrelations between the identified factors or latent variables. Overall, they suggest that it is a consistent scale, as discussed in the reliability calculation, with non-orthogonal factors, meaning they are related to each other.

The arrows originating from each factor towards the items that compose them (observed variables or indicators) show the regression coefficients between each question and the contributing factor. This provides information about the weight each item has within that factor or dimension. All coefficients are positive, indicating a positive contribution (higher value, greater contribution) for each of them.

Finally, the values on the left side of the figure, associated with each item, indicate the variability of responses to each question, measured by the variance. It is important for the variability to remain at intermediate values, as obtained in this questionnaire, because very high variance would be associated with greater error, while very small or zero variance implies a lack of reliability and, therefore, a lack of validity. Two additional questions related to pain, included in the Picker questionnaire due to their relevance in daily practice, were added to these 17 questions that fit the model (Figure 2, File S1).

**Figure 4:**
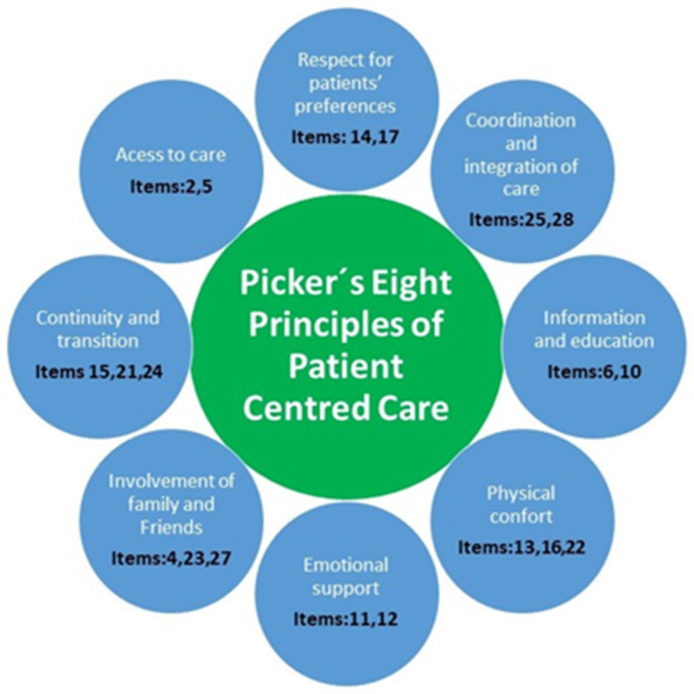
Questionnaire items framed in the 8 standards that represent the conceptual framework.

In Figure 4, the items framed in the 8 standards that represent the conceptual framework are shown.

## 4. Discussion

This work develops and validates a questionnaire that allows us to measure patient experience of hospital nursing care received in a holistic way. This questionnaire may be used in order to identify improvements referred to hospital nursing care and as a leadership and management instrument of nursing teams. Additionally, it will provide useful information to assess the organization’s culture and to evaluate and design healthcare processes to respond, as much as possible, to the needs, circumstances, and environment of patients [3].

There are various questionnaire models that assess patient experience in different cultural contexts, such as the Consumer Assessment of Healthcare Providers and Systems (CAHPS), Picker Patient Experience Questionnaire, Quality from the Patients’ Perspective (QPP), and QPP-Shortened (QPPS), Patient Experience Questionnaire (PEQ), The Nordic Patient Experience Questionnaire (NORPEQ), and Hong Kong Inpatient Experience Questionnaire (HKIEQ), but none are exclusively focused on nursing care [15,16,17,18,19,20].

The Picker Patient Experience Questionnaire was chosen as the foundation for its development because it has several advantages: It is explicitly designed to measure the patient experience, can be supplemented with additional questions to expand the study, and can be systematically included in other surveys without compromising its validity (Supplementary Materials). It allows for international comparisons, is translated and validated in Spanish, and has been used in the Spanish context [11,12].

This new questionnaire includes the eight aspects that shape the patient experience [21]. (1) Respect for patient-centered values (e.g., item 14); (2) coordination and integration of care (e.g., item 25); (3) information, communication, and education on clinical status, self-care, and health promotion (e.g., item 10); (4) physical comfort including pain management, help with activities of daily living, and clean and comfortable surroundings (e.g., item 16); (5) emotional support and alleviation of fear and anxiety about such issues as clinical status (e.g., item 11); (6) welcoming the involvement of family and friends and accommodation of their needs as caregivers (e.g., item 4); (7) transition and continuity as regards information that will help patients care for themselves away from a clinical setting, and coordination, planning, and support to ease transitions (e.g., item 21); (8) access to care with attention (e.g., item 2).

It is essential to consider that instruments should be chosen, in addition to their psychometric properties, based on the purpose and context of the survey [22]. This underscores the need for countries to develop valid and reliable instruments specific to their context to accurately capture aspects of care important to the population they serve. The statistical analysis verified the reliability and validity of the questionnaire, showing adequate psychometric properties for its application in nursing care in the hospital setting [23].

Finally, the 17 items were grouped into the following aspects: interrelationships—11, 22, 25; nursing care—2, 4, 5, 14; information during hospital stay—6, 10, 12; patient’s rights information—17, 24; and discharge information—15, 21, 23, 28. A total of two items related to pain were added to these dimensions. To these dimensions, two items on pain (item 13 and 16) were added, given the importance of the topic considered by the expert panel and because it is one of the eight aspects that shape the patient experience [21].

This tool has been designed to evaluate the patient’s experience, prioritizing characteristics in its design, such as a manageable number of items for accessibility, easy comprehension, and a focus on patient experience related to nursing care. These characteristics follow the model of the work conducted by José Joaquín Mira and colleagues [24] in the validation of the patient experience assessment questionnaire for chronic diseases.

### 4.1. Limitations, Strengths, and Future Research

One limitation of the study is that it has only been administered in one hospital and is not valid for certain units such as emergency and psychiatry. In order to further enhance the applicability and robustness of our developed questionnaire, future research directions should include validation efforts across diverse hospital settings and exploration of its suitability in various clinical contexts.

However, a strength is the availability of a sufficient sample size and a high response rate. The response rate was high, possibly due to the procedure in which the questionnaire was conducted—face-to-face by the nurse, who recorded the patient’s responses after asking the questions. Considering that survey response rates tend to be highly variable and often low [23], we believe this aspect has been one of the strengths of the study.

### 4.2. Implications for Healthcare Improvements

Our questionnaire provides feedback on the care process, focusing on effects such as communication with healthcare professionals, provided information, involvement in decisions, physical well-being, emotional support, and care transitions [25].

## 5. Conclusions

A questionnaire has been crafted to address a key healthcare need, specifically focusing on the evaluation of patients’ experience of nursing care in the hospital setting. Going forward, the implemented questionnaire not only offers a valuable tool for identifying areas of improvement in nursing care, but also has the potential to have a significant impact on healthcare quality improvement initiatives. Its focus on patient-centered values, care coordination, and other critical dimensions positions it as a valuable asset to healthcare organizations seeking to improve the patient experience and overall quality of care.

## Figures and Tables

**Table 1:** Sample characteristics (N, %).

Sociodemographic Variables		N	%
Sex	Woman	79	50%
	Man	79	50%
Age (years)	<56	41	25.9%
	56–70	44	27.8%
	70–81	39	24.7%
	≥82	34	21.5%
Marital Status	Single	21	13.3%
	Living with a partner/Married	84	53.2%
	Divorced/Separated	22	13.9%
	Widowed	31	19.6%
Education level	No formal education	19	12%
	Primary	71	44.9%
	Secondary	48	30.4%
	University	20	12.7%
Occupational Status	Student	2	1.3%
	Self-employed	8	5.1%
	Salaried employee	22	13.9%
	Retired	98	62%
	Unemployed	13	8.2%
	Homemaker	15	9.5%

**Table 2:** Kappa agreement coefficient between measures at hospital discharge and 1 month later.

Item	Kappa	Asymptotic Standard Error	Student’s t	*p*
1	0.244	0.105	2.675	0.007
2	0.272	0.103	2.784	0.005
3	0.215	0.137	2.130	0.033
4	0.376	0.106	3.826	0.000
5	0.225	0.126	2.368	0.018
6	0.123	0.091	1.414	0.157
7	0.261	0.088	3.329	0.001
8	0.357	0.115	3.696	0.000
9	0.167	0.140	1.861	0.063
10	0.290	0.124	3.064	0.002
11	0.603	0.164	5.550	0.000
12	0.022	0.097	0.245	0.807
13	0.481	0.127	3.384	0.001
14	0.013	0.083	0.236	0.813
15	0.147	0.100	1.679	0.093
16	0.458	0.108	3.749	0.000
17	0.104	0.126	1.016	0.310
18	0.492	0.105	4.979	0.000
20	0.657	0.319	4.847	0.000
21	0.361	0.093	3.596	0.000
22	−0.063	0.027	−0.518	0.605
23	0.010	0.093	0.108	0.914
24	0.043	0.131	0.407	0.684
25	0.100	0.111	0.937	0.349
26	0.143	0.175	1.505	0.132
27	0.309	0.107	3.337	0.001
28	0.173	0.092	2.088	0.037
29	−0.092	0.091	−0.981	0.327

**Table 3:** Simplified factor loading matrix.

Item	Component				
	1	2	3	4	5
2		0.698			
4		0.711			
5		0.574		0.470	
6				0.554	
8					
9					0.429
10				0.509	0.570
11	0.588				
12				0.468	
14		0.543			
15			0.346		
17			0.733		
18		0.350			
20	0.987				
21			0.612		
22	0.783				
23			0.402		
24			0.646		
25	0.730				
27		0.638			
28					0.614
29	0.347				

Note: Factor loadings below 0.30 have been omitted.

**Table 4:** Fit indicators of data to the model.

Goodness of Fit Statistics and Reference Criteria		
Statistics	Abbreviation and Obtained Value	Criteria
Fit Statistics:		
Chi-square	Χ^2^ = 136.54; *p* = 0.03819	*p* > 0.01 *
Chi-square/degrees of freedom ratio	Χ^2^/g.l. =136.54/109 = 1.253	<3 **
Comparative fit:		
Comparative Fit Index (CFI)	CFI = 0.98	≥0.95 **
Tucker–Lewis Index (TLI)	TLI = 0.98	≥0.95 **
Standardized Root Mean Square Residual (SRMR)	SRMR = 0.92	≥0.90 *
Parsimonious fit:		
Parsimony Adjusted Normed Fit Index (NFI)	NFI = 0.74	Close to 1 *
Other:		
Goodness-of-fit Index (GFI)	GFI = 0.96	≥0.95 **
Adjusted Goodness-of-fit Index (AGFI)	AGFI = 0.94	≥0.90 **
Root Mean Square Residual (RMSR)	RMSR = 0.091	Close to 0 **
Root Mean Square Error of Approximation (RMSEA)	RMSEA = 0.040	<0.080 **

Note: * acceptable fit; ** good fit.

## Data Availability

Dataset available on request from the authors.

## Supplementary Materials

The following supporting information can be downloaded at: https://www.mdpi.com/article/10.3390/nursrep14010031/s1, File S1: Original questionnaire administered to the patient; list of items conforming the final questionnaire.

## Public Involvement Statement

No public involvement in any aspect of this research.
