# Supplementary material for: Design and Validation of a Questionnaire to Measure Patient Experience in Relation to Hospital Nursing Care
Source: Nurs Rep. 2024 Feb 9;14(1):400–12. doi: 10.3390/nursrep14010031 (PMC10885121; doi:10.3390/nursrep14010031)
Supplement: Supplementary file 1 [file nursrep-14-00031-s001.zip › nursrep-2814043-File S1.pdf]

QUESTIONNAIRE S1. ORIGINAL QUESTIONNAIRE ADMINISTERED TO THE PATIENTS

1. Do you know the Charter of Patient Rights and Responsibilities?
- Yes, totally - No
- Yes, at some extent - DK/NR

2. Did the nursing team professionals identify themselves when entering your room to make any intervention?
- Yes, totally - No
- Yes, at some extent - DK/NR

3. Did you want to be more involved in the decision making of your acre and treatment?
- Yes, totally - No
- Yes, at some extent - DK/NR

4. Did the nursing team take care of you and your family/relatives to help you and teach you self-care?
- Yes, totally - No
- Yes, at some extent - DK/NR

5. Did the nurse spend enough time when informing you about your illness related care?
- Yes, always - No
- Yes, sometimes - No, gave no information
- DK/NR

6. Did the nurse explained all you wanted to know about treatment effects?
- Yes, totally - No
- Yes, at some extent - DK/NR

7. When the nurse explained to you your illness aspects, did she try to avoid other patients from hearing her (e.g. spoke in quiet voice, drawing separation curtain)?
- Yes, always - I was in a single room
- Sometimes
- No - DK/NR

8. Did the nursing team members address you by your name?
- Yes, always - No
- Sometimes - DK/NR

9. The nursing team, did help your hospital stay to be more bearable in the absence of your family or relatives?
- Yes, totally - No

- Yes, at some extent - DK/NR

10. When you had questions, did you get clear and easy to understand answers from nursing staff?

– Yes, always - I had no questions

- Yes, at some extent - DK/NR
- No

11. Do you consider the relation with the nursing team close?

- Yes, totally - No

- Yes, at some extent - DK/NR

12. Did the nurses encouraged you to ask about your illness or any other aspect related to it?

- - Yes, totally - No
- Yes, at some extent - DK/NR

13. Did you feel pain at some stage?

-Yes -No - DK/NR

14. Did the nursing team try to protect your intimacy while admitted in the hospital? (e.g. when examinations were carried out, bed bathing, going to the toilet or change of clothes)

- Yes, totally - Not needed as I could do everything by myself
- At some extent - DK/NR
- No

15. Did the nurse explain in a comprehensive way what the medications you had to have at home were for?

- Yes, totally - Didn´t need explanations
- Yes, at some extent - Didn´t have to take medications
- No - DK/NR

16. Do you think the nurse did as much as she could to relief your pain?

- Yes, totally - No
- Yes, at some extent - DK/NR

17. Did the nurse informed you about your right to reject treatments or tests?

- Yes, totally - No
- Yes, at some extent - DK/NR

18. Were you able to identify the different members of the healthcare team?

- Yes, totally - No, I couldn´t differentiate team members who took care of me
- Only physicians who took care of me - DK/NR
- Only nurses who took care of me

19. At any time during your admission, did the nursing team commented on issues not related to you in front of you?

- Yes, very often - No
- Sometimes - DK/NR

20. In general, did you feel treated with respect by the nursing team during your stay in the hospital?

– Yes, always - No
- Sometimes - DK/NR

21. Did the nurse talk to you about the warning signs related to your disease or treatment which you should be careful about?

- Yes, totally - No
- Yes, at some extent - DK/NR

22. Did the nursing team look after your comfort and facilitate your rest?

- Yes, always - No
- Sometimes - DK/NR

23. Did the nurse provide all the necessary information to your family or relatives to help with your recovery?

- Yes, totally - my family didn´t need or want any information
- At some extent - I didn´t want any of my next of kin or friends talk to the nurse
- No
- There were no family members or relatives involved - DK/NR

24. Did the nurse inform you about your right to know about what is written in your medical record?

- Yes, totally - No
- Yes, at some extent - DK/NR

25. Did you feel the nursing team was working in team and was well organized?

- Yes, always - No
- Sometimes - DK/NR

26. Did the nurse verify your identity (name, room number, bed…) before giving medication, blood withdrawal…?

- Yes, always - No
- Sometimes - DK/NR

27. Did the nursing team show interest for your family or relatives' comfort?

- Yes, always - No
- Sometimes - DK/NR

28. Did the nurse explain the possible side effects of the medication that you should take into account?

- Yes, totally - Didn´t need explanations
- At some extent - Didn't have to take any medication
- No - DK/NR

29. Did the nurse explained anything you wished to know about any type of care/techniques provided to you?

QUESTIONNAIRE S2. THE LIST OF ITEMS CONFORMING THE FINAL QUESTIONNAIRE

Interrelationships: 11, 22, 25

- Do you consider the relation with the nursing team close?
- Did the nursing team look after your comfort and facilitate your rest?
- Did you feel the nursing team was working in team and was well organized?

Nursing care: 2, 4, 5, 14, 27

- Did the nursing team professionals identify themselves when entering your room to make any intervention?
- Did the nursing team take care of you and your family/relatives to help you and teach you self-care?
- Did the nurse spend enough time when informing you about your illness related care?
- Did the nursing team try to protect your intimacy while admitted in the hospital? (e.g. when examinations were carried out, bed bathing, going to the toilet or change of clothes)
- Did the nursing team show interest for your family or relatives' comfort?

Information during hospital stay: 6, 10, 12

- Did the nurse explained all you wanted to know about treatment effects?
- When you had questions/ concerns, did you get clear and easy to understand answers from nursing staff?
- Did the nurses encouraged you to ask about your illness or any other aspect related to it?

Patient's rights information: 17, 24

- Did the nurse informed you about your right to reject treatments or test?
- Did the nurse inform you about your right to know about what is written in you medical record?

Discharge information: 15, 21, 23, 28

- Did the nurse explain in a comprehensive way what the medications you had to have at home were for?
- Did the nurse talk to you about the warning signs related to your disease or treatment which you should be careful about?
- Did the nurse provide all the necessary information to your family or relative to help with your recovery?
- Did the nurse explain the possible side effects of the medication that you should take into account?

Pain related: 13, 16

- Did you feel pain at some stage?
- Do you think the nurse did as much as she could to relief your pain?
